# How to manage falls in hospitalized patients: A single center experience in allogeneic stem cell transplantation setting

**DOI:** 10.1097/MD.0000000000029132

**Published:** 2022-03-18

**Authors:** Marco Cioce, Franziska Michaela Lohmeyer, Stefano Botti, Elena Rostagno, Laura Orlando, Giuseppe Vetrugno, Paolo Oppedisano, Maurizio Zega, Simona Sica, De Stefano Valerio, Andrea Bacigalupo, Alberto Fiore

**Affiliations:** ^a^ *UOC SITRA, Fondazione Policlinico Universitario A. Gemelli IRCCS, Rome, Italy,* ^b^ *Direzione Scientifica, Fondazione Policlinico Universitario A. Gemelli IRCCS,* *Rome, Italy,* ^c^ *Hematology Unit, Azienda USL-IRCCS of Reggio Emilia, Italy,* ^d^ *UO* *Pediatria - Prof. A. Pession Programma di Oncologia Ematologia e Terapia* *Cellulare IRCCS AOU, Bologna, Italy,* ^e^ *Bone Marrow Transplant Unit, Oncology* *Institute of Southern Switzerland, Bellinzona, Switzerland,* ^f^ *UOS Risk* *Management, Fondazione Policlinico Universitario A. Gemelli IRCCS, Rome, Italy,* ^g^ *UOS Qualità e Accreditamento - Direzione Sanitaria, Fondazione Policlinico* *Universitario A. Gemelli IRCCS, Rome, Italy,* ^h^ *Istituto di Ematologia, Università* *Cattolica del Sacro Cuore, Fondazione Policlinico Universitario Gemelli, Rome, Italy.*

**Keywords:** allogeneic hematopoietic cell transplantation, fall, root cause analysis, thrombocytopenia

## Abstract

In allogeneic hematopoietic stem cell transplantation (AHSCT), falls can lead to immediate and late consequences and in some cases to death. We analyzed risks and causes of falls with root cause analysis (RCA) based on which improvement interventions were implemented.

A retrospective observational study was conducted to analyze with RCA data of incidence reports and medical records of patients admitted; an expert panel identified actions to prevent falls, which were collected in a checklist.

Between December 2017 and November 2019, 214 patients were admitted to ordinary hospital stays for AHSCT or AHSCTrelated complications. In this period, 15 falls, involving 11 patients, occurred resulting in a 2.32 d/patient incidence. In 66.67% of cases minor head trauma occurred. Diuretic drugs (93.33%), induced hyper-diuresis in nonbladder catheter patients (93.33%) and antihypertensive drugs (93.33%) were reported as most common cause in our incident reports. The most frequent fall time slot was between 10 PM and 7 AM (60%). We determined with RCA diuretics and consequent induced hyper-diuresis (80%), self-insufficiency (40%), antihypertensive (33.3%) and noncompliance (33.3%) as the most common cause of falls. Finally, 16 actions, collected in a “safe comfort” checklist, were identified to prevent falls.

Diuretic drugs inducing hyper-diuresis, self-insufficiency, poor patient compliance, orthostatic hypotension, fever, night-time and obstacles within inpatient units are the most common contributing factors. Therefore, administration of diuretic and antihypertensive drugs should be rescheduled and a multidimensional risk assessment scale integrated with a preventive action plan, such as the safe comfort checklist, should be implemented to reduce falls.

## 1. Introduction

Falls are among the most common adverse events in health care facilities, both in hospital and outpatient settings affecting usually frail patients, many with dementia.^[1]^ Some studies report incidences ranging from 2 to 20 falls per 1.000 d/patients.^[2,3]^

About one third of over 65 years old patients fall at least once a year. About 10% of falls cause serious damage, including skin excoriation, fractures, dislocations, and head trauma.^[4]^ Several factors, such as age, gender, comorbidity, physical and psychosocial dysfunction, and medications increase the risk of falls; these factors affect both the general population and elderly.^[5,6]^

In addition to physical and psychological damage, falls in the hospital setting increase patients’ hospital stay requiring additional diagnostic and therapeutic activities and/or any further hospitalizations after discharge, which leads to an increase in health and social costs.^[7]^ In fact, according to the medical malpractice report on the trend of medical malpractice risk in the Italian public and private healthcare sector, 9.9% of damage claims, from 2009 to 2017, are due to accidental falls in hospitalized patients or during outpatient care, which cost almost 33 million euros. Falls are the fourth frequent damage claim type after surgical, diagnostic and therapeutic error and 90% of falls are avoidable. According to the medical malpractice report, damage claims for falls were correlated in 97% of cases to injuries and 2.4% of deaths.^[8]^

About 14% of hospital falls can be classified as accidental determined by environmental factors (eg, slipping on the wet floor), 8% as unpredictable, given the physical condition of the patient (eg, sudden disturbance of balance), and 78% as predictable.^[9]^ The prediction of falls which fall risk assessments can have beneficial implications in hospital practice and reduce medical-legal litigation,^[2]^ specifically if operators, patients and family/caregivers gain awareness of fall risks.^[10]^

The risk of falling, although always present, is different in various hospital settings; in particular, in allogeneic hematopoietic stem cell transplantation (AHSCT) falls can lead to serious immediate or late consequences and in some cases even to death,^[10]^ high mortality and morbidity are still attributed to AHSCT. In fact, the first few weeks after hematopoietic stem cells infusion are associated to toxic effects due to treatment with chemo and/or high-dose radiotherapy and severe pancytopenia resulting from the suppression of the patient’s hematopoietic system. Particularly in this first phase, patients are at high risk of hemorrhagic complications.^[11]^ In fact, thrombocytopenia induced by the pretransplant conditioning regimen can cause potential fatal outcomes if a fall occurs. Numerous clinical conditions, such as hypotension, febrile neutropenia, anemia, fatigue, and pharmaceuticals such as antiepileptic drugs, antihypertensive, diuretics increase the risk of falling.^[12]^ Patients frequently fall close after stem cells infusion, especially in the preengraftment phase. Despite various prevention efforts, falls remain a serious safety problem.^[13]^

Considering the potential serious consequences of a fall in AHSCT patients, we analyzed risks and causes of falls with root cause analysis (RCA) based on which improvement interventions were implemented supported by appropriate tools.

## 2. Materials and methods

### 2.1. Study design, patients and setting

A retrospective observational study was conducted analyzing data from medical records and incident reports (IR) of patients admitted at our hospital (central Italy) for AHSCT or AHSCTrelated complications who fall during their hospital stay. This study examined clinical data from 291 hospitalizations between December 2017 and November 2019 involving 214 patients over 16 years. The Conley scale was used to assess the risk of accidental falls in patients.

The study was approved by the local ethics committee on February 11, 2021 (ID: 3753).

### 2.2. Root cause analysis

To analyze causes and factors related to falls during hospitalization (ie, adverse event), RCA was used according to the model proposed by the Canadian Patient Safety Institute,^[14]^ to analyze all cases based on which actions to improve patient safety during their hospital stay were developed.^[15]^ The Nominal Group Technique was used to easily, neutrally and anonymously reach consensus among RCA group participants on the priorities of contributing factors and root causes and to propose improvement actions to be implemented.^[16]^

An expert group (risk manager, AHSCT center coordinator and 3 nurses) analyzed in a preliminary meeting all falls of the respective timeframe based on information of the IR (eg, dichotomous evaluation of self-sufficiency) and computerized medical records. During brainstorming after 7 days, for each single IR, one or more fall causes were listed on post-it sheets by each expert. The group leader collected all post-it notes, that is, all causes for falling proposed by each team member, which were validated on the next day by all experts with a 4 point Likert scale (4 = most significant, 1 = less significant). An Ishikawa cause and effect diagram was created to visualize contributing factors and root causes.

### 2.3. The “safe comfort” checklist

To prevent accidental falls in patients at the AHSCT unit, we developed a “Safe Comfort” checklist. Several items from different references and operational sources (RCA, regional documents, standard operating procedures, etc) were collected. A panel of 6 Philosophiae Doctor evaluated relevance, clarity, simplicity, and ambiguity and content validity index (CVI) was calculated for each item (I-CVI) and for the checklist as a whole (S-CVI).^[17,18]^

Once validated, nurses with at least 3 years of experience in the AHSCT, part of the Italian group for bone marrow transplantation, hematopoietic stem cells and cell therapy network, rated online with a Delphi method checklist items with a 4 point Likert scale (not relevant to very relevant). The sample size of validators (63 recruited individuals, considering possible dropouts) was calculated by looking at a repeating-sized ANOVA model, with alpha -0.01 and power 80% a delta of 0.6325 over 2 groups; a variance between groups of 0.0200 and a variance explained by effect between-within groups -0.05 for 2 or more repeated measures with a correlation rho -0.9. At least 51% of the responses corresponding to a score of 3 or 4 on the Likert scale,^[19]^ an interquartile gap less than 1^[20,21]^ and a standard deviation (SD) less than 1.5^[22]^ indicated absolute consensus among experts. Finally, reliability of internal consistency of the checklist was calculated with Cronbach alpha coefficient.

### 2.4. Data collection and statistical analysis

Descriptive statistics were used to describe the sample techniques. Quality variables were described using absolute frequencies and percentages, while quantitative variables were summarized through range, average, median, and SD values. The normality of the values was verified with the Shapiro-Wilk test. Comparisons were calculated with *t* tests for matched data or Kruskal-Wallis and ANOVA. The data was stored and managed in spreadsheets (data set built on spreadsheet type Microsoft Excel 2016 for Mac Vers. 2016/14.5.5). Statistical analyses were performed through Stata (StataCorp LLC)/IC software (14.2 for Mac ([4-bit Intel], Vers. January 9, 2017, 800-STATAPC - Lakeway). Statistical significance has been set at *P* < .05.

## 3. Results

### 3.1. Descriptive statistics

Two hundred fourteen patients, between 16 and 74 years, were admitted to ordinary hospital stays for AHSCT or treatmentrelated complications. In the period described, 15 falls involving 11 patients with an incidence of 2.32 d/patient occurred.

We observed an average age of 48.84 (SD 13.99) years in all admitted patients. In patients who fallen average age was 58.64 (SD 11.4) years, men 66 (SD 6.1) years and women 50 (SD 10.8) years (*P* = .028); and in patients who did not fallen 48.31 (SD 13.94) years (*P* = .037); the average stay was 23.7 (SD 22.1) days in all patients. Instead, in patients who fallen average stay was 63.64 (SD 27.8) days, men 55 (SD 12.28) days, women 74 (SD 38.3) days (*P* = .061). In patients who did not fall average stay was 22.14 (SD 20.36) days (*P* = .0001) (Table 1).

**Table 1:** AHSCT patients’ characteristics.

**Variables**	**Total**	**Fall**	**No fall**	***P*-value**
Patients	214	11	203
Mean age (SD) yr	48.84 (13.99)	58.64 (11.4)	48.31 (13.94)	**.037**
Male gender (n, %)	129 (60.28%)	6 (54.55%)	123 (60.59%)	.756^*^
Female gender (n, %)	85 (39.72%)	5 (45.45%)	80 (39.41%)
Admissions	291	11	280
Mean hospitalization (SD) d	23.7 (22.9)	63.64 (27.8)	22.14 (20.36)	**.001**
AHSCT = allogeneic hematopoietic stem cell transplantation, SD = standard deviation.				

^*^Pearson chi-square test.

Of the 11 patients who had fallen, 8 (72.73%) fell 1 time, 2 (18.18%) fell 2 times, and 1 (9.09%) fell 3 times. In 80% of the 15 events, the risk of falling was assessed with the Conley scale (time of first assessment mean 6.8 days from hospital admission); of these, 9 (75%) were defined as at risk (Conley score 2) and 3 (25%) patients as not at risk (Conley score 1) (Table 2).

**Table 2:** Data on falls and fall risk assessment.

		**N (%)**	**Mean (SD)**	**95% CI**
Patients who fall		11
	Once	8 (72.7)
	Twice	2 (18.2)
	Three	1 (9.1)
Number of falls		15
Conley risk assessment (total)		12 (80)
	Conley score ≥ 2	9 (75)
	Conley score ≤ 1	3 (25)
	Conley score		3.4 (2.23)	1.98-4.82
	Days from admission to risk assessment		6.8 (8.19)	1.6-12
Conley scale sensitivity		(75%)		0.46-0.91
95% CI = 95% confidence intervals, SD = standard deviation.				

The average post-transplant discharge was 44 (SD 24.0) days in men 37 (SD 21.4) days and in women 52 (SD 26.6) days (*P* = .061). The adverse event occurred on average 24 (SD 16.1) days after stem cells infusion.

More than half (53.33%) of the patients who fell had acute myeloid leukemia receiving HSCT from haploidentical family members (46.67%). In 66.67% of cases a minor head trauma occurred and none of the events analyzed caused long-term consequences or serious injury.

Among the most common baseline causes reported in IR, we found the use of diuretic drugs (93.33%) induced hyper-diuresis in nonbladder catheter patients (93.33%) and the use of antihypertensive drugs (93.33%) (Table 3). The most frequent fall time slot was between 10:00 PM and 7:00 AM in 60% of patients (Fig. 1).

**Table 3:** Fall characteristics, fall-related outcomes, and causes (N = 15).

		**Freq.**	**%**
Pathology	
	AML	8	53.33
	ALL	5	33.33
	Ly	1	6.67
	MDS/MPS	1	6.67
Transplant type	
	HLA Id. Sib.	1	6.67
	Unrelated donor	5	33.33
	Family Mismatched/Aplo	7	46.67
	Other	2	13.33
Serious consequences	
	Yes	0	0
	No	15	100.00
Head trauma	
	Yes	5	33.33
	No	10	66.67
Degree of injury	
	Absent	7	46.67
	Mild	6	40.00
	Moderate	2	13.33
Bladder catheter	
	Yes	1	6.67
	No	14	93.33
Diuresis mL/d	
	<2.500	5	33.33
	2.501-3.000	2	13.33
	3.001-3.500	1	6.67
	3.501-4.000	1	6.67
	4.001-4.500	3	20.00
	>4.500	3	20
Antihypertensive drugs	
	Yes	14	93.33
	No	1	6.67
Diuretic drugs	
	Yes	14	93.33
	No	1	6.67
ALL = acute lymphocytic leukemia, AML = acute myeloid leukemia, Aplo = Haploidentical, HLA = Human Leukocyte Antigen, Id. Sib = Identical Sibling, Ly = lymphoma, MDS/MPD = myelodysplastic syndromes/myeloproliferative diseases.			

**Figure:**
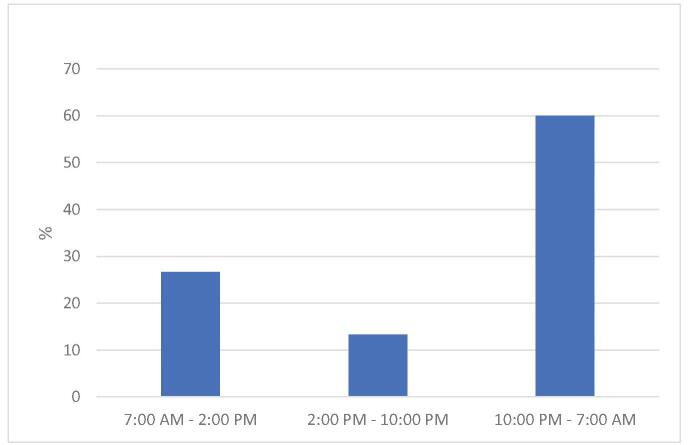
**Figure 1.** Time slot of fall occurrence.

### 3.2. Root cause analysis

In 4 of the 15 IR analyzed, root causes of accidental fall were not identifiable due to lack of absolute consent among the experts; where present, the most frequent root causes were diuretics and consequent induced hyper-diuresis (80%), incomplete self-sufficiency (40%), antihypertensive (33.3%) and noncompliance (33.3%) (Fig. 2). Figure 3 shows the Ishikawa diagram describing contributing factors and root causes.

**Figure:**
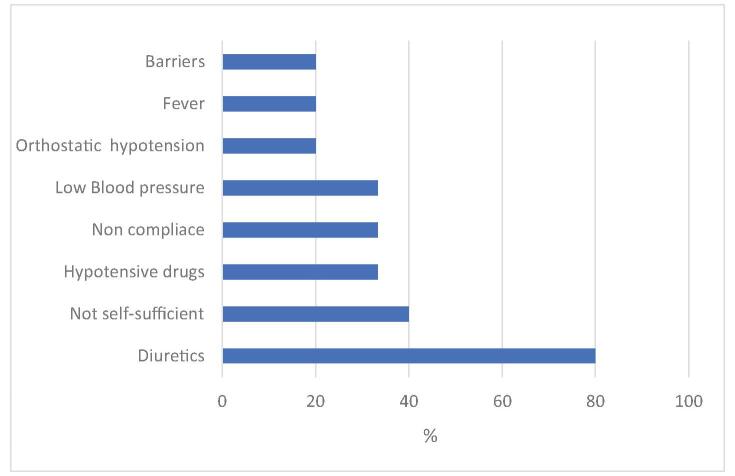
**Figure 2.** Main results of the route cause analysis: frequent fall causes.

**Figure:**
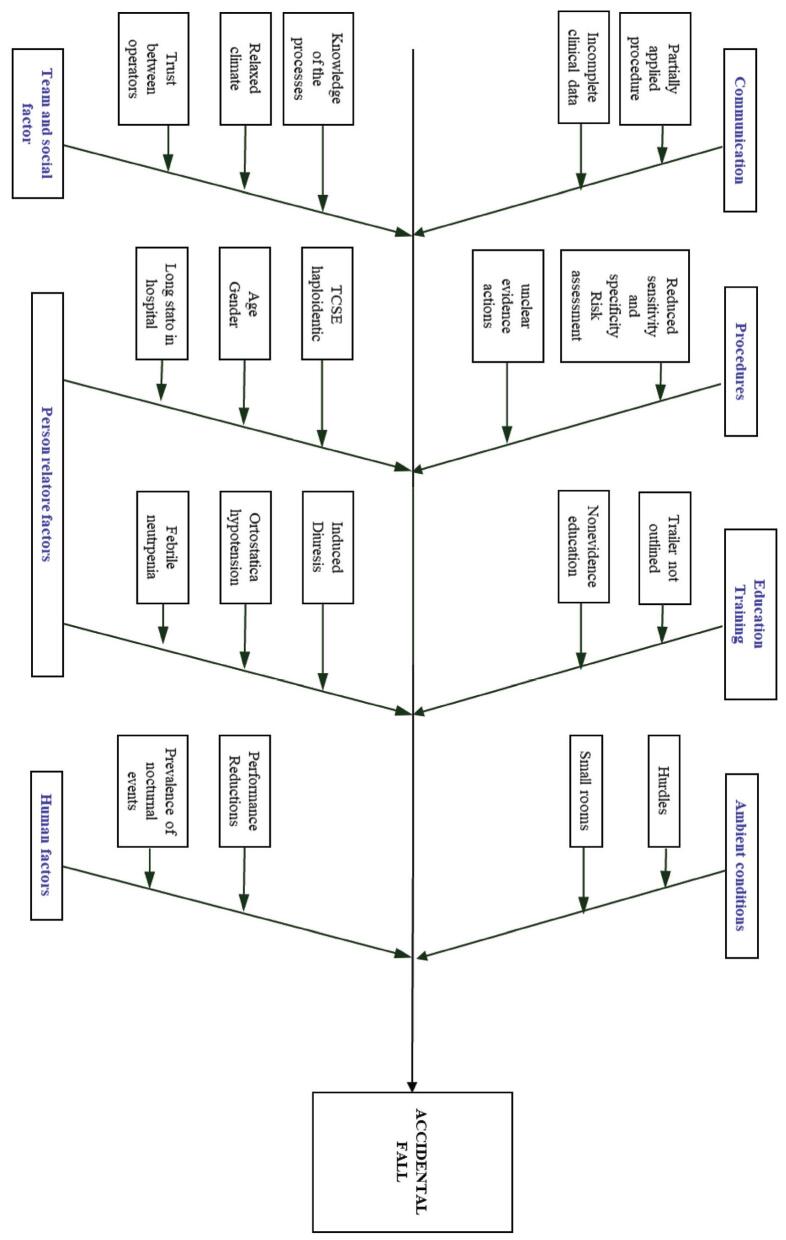
**Figure 3.** Ishikawa diagram: falls, contributing factors/root causes.

Among the improvement actions identified, in addition to retraining nursing staff and reorganization of the administration of diuretic and antihypertensive drugs, the “Safe Comfort” checklist was implemented at our AHSCT unit. Of the initially 25 items proposed to the group of 6 experts, 3 were removed due to an I-CVI < 0.83 (I23: 0.71; I24: 0.58; I25: 0.54); S-CVI was 0.93. The remaining 22 items were submitted to the group of 63 nurses who identified 16 actions deemed effective to prevent falls, which finally constitute the list of items of the “Safe Comfort” checklist (Table 4). The reliability of the internal consistency of the checklist measured with Cronbach alpha coefficient was 0.80.

**Table 4:** Actions to reduce the risk of falling in hospitalized patients (Pre-“safe comfort” checklist).

			**I-CVI**	**Total I-CVI**	**Mean**	**SD**	**IQR**	**% 3-4**
I1	Bed wheel lock	Relevance	1.00	1.00	4.00	0.20	0.00	100.00
		Simplicity	1.00
		Clarity	1.00
		Ambiguity	1.00
I2	Accessible nurse call cable	Relevance	1.00	1.00	3.90	0.30	0.00	100.00
		Simplicity	1.00
		Clarity	1.00
		Ambiguity	1.00
I3	Obstacle-free surroundings	Relevance	1.00	1.00	3.90	0.30	0.00	100.00
		Simplicity	1.00
		Clarity	1.00
		Ambiguity	1.00
I4	Patient instruction: Invitation to get up slowly after spending a few minutes sitting on the bed	Relevance	1.00	1.00	3.90	0.30	0.00	100.00
		Simplicity	1.00
		Clarity	1.00
		Ambiguity	1.00
I5	Patient education: invitation not to walk on wet floor	Relevance	1.00	1.00	3.90	0.40	0.00	98.41
		Simplicity	1.00
		Clarity	1.00
		Ambiguity	1.00
I6	Patient education: invitation to use appropriate clothing and shoes	Relevance	1.00	1.00	3.90	0.50	0.00	98.41
		Simplicity	1.00
		Clarity	1.00
		Ambiguity	1.00
I7	Patient education: concrete description of fall risk	Relevance	1.00	1.00	3.80	0.40	0.00	100.00
		Simplicity	1.00
		Clarity	1.00
		Ambiguity	1.00
I8	Patient education: Invitation to ask for help during small trips or to go to the bathroom	Relevance	1.00	1.00	3.80	0.40	0.00	100.00
		Simplicity	1.00
		Clarity	1.00
		Ambiguity	1.00
I9	Patient instruction: Demonstration of nurse call cable in bathroom	Relevance	1.00	1.00	3.80	0.50	0.00	96.83
		Simplicity	1.00
		Clarity	1.00
		Ambiguity	1.00
I10	Patient education: invitation to use glasses in case of visual impairment	Relevance	1.00	1.00	3.80	0.40	0.00	96.83
		Simplicity	1.00
		Clarity	1.00
		Ambiguity	1.00
I11	Providing info-educational material	Relevance	1.00	1.00	3.70	0.50	0.00	95.24
		Simplicity	1.00
		Clarity	1.00
		Ambiguity	1.00
I12	Patient education: Holding tightly to an object when bowing	Relevance	1.00	1.00	3.70	0.70	0.50	95.24
		Simplicity	1.00
		Clarity	1.00
		Ambiguity	1.00
I13	Patient education: invitation to urinate sitting or to use the parrot	Relevance	1.00	1.00	3.70	0.50	0.50	96.83
		Simplicity	1.00
		Clarity	1.00
		Ambiguity	1.00
I14	Accessibility to frequently used items (smartphones, handkerchiefs, thermometer, vomit bag, etc)	Relevance	1.00	1.00	3.70	0.50	0.50	96.83
		Simplicity	1.00
		Clarity	1.00
		Ambiguity	1.00
I15	Minimum-height bed position whenever possible	Relevance	1.00	1.00	3.70	0.60	0.50	93.65
		Simplicity	1.00
		Clarity	1.00
		Ambiguity	1.00
I16	Family/caregiver involvement	Relevance	1.00	1.00	3.60	0.60	0.50	96.83
		Simplicity	1.00
		Clarity	1.00
		Ambiguity	1.00
I17	Turning on night light	Relevance	1.00	1.00	3.40	0.80	1.00	85.71
		Simplicity	1.00
		Clarity	1.00
		Ambiguity	1.00
I18	Incontinence pants to manage fecal and/or urinary incontinence: comfortable chair	Relevance	0.83	0.83	3.10	0.90	1.00	77.78
		Simplicity	0.83
		Clarity	0.83
		Ambiguity	0.83
I19	Bed bank usage	Relevance	0.83	0.96	3.10	0.90	2.00	73.02
		Simplicity	1.00
		Clarity	1.00
		Ambiguity	1.00
I20	Device to manage urinary incontinence/urgency: uroprophylactic	Relevance	1.00	0.88	3.00	1.00	1.00	76.19
		Simplicity	0.83
		Clarity	0.83
		Ambiguity	0.83
I21	Device to manage urinary incontinence/urgency: bladder catheters	Relevance	0.83	0.83	2.80	0.90	1.50	63.49
		Simplicity	0.83
		Clarity	0.83
		Ambiguity	0.83
I22	Device to manage urinary incontinence/urgency: diaper	Relevance	0.83	0.96	3.00	1.00	2.00	69.84
		Simplicity	1.00
		Clarity	1.00
		Ambiguity	1.00
I23	Patient instruction: invitation to go to the bathroom accompanied, at regular intervals	Relevance	0.67	0.71
		Simplicity	0.83
		Clarity	0.67
		Ambiguity	0.67
I24	Device to manage diarrhea: rectal probe	Relevance	0.50	0.58
		Simplicity	0.67
		Clarity	0.67
		Ambiguity	0.50
I25	Use half-bed long banks	Relevance	0.33	0.54
		Simplicity	0.50
		Clarity	0.67
		Ambiguity	0.67
S-CVI = 0.93. CVI = content validity index, IQR = Interquartile range, SD = standard deviation.								

## 4. Discussion

In 291 admissions surveyed, we recorded an incidence of 2.32 falls per 1000 d/patients, which is not higher than falls reported in literature for the general population.^[2,3]^ Similar to Miwa et al,^[12]^ the median age of patients who fall was around 60 years while the most frequent pathology was acute myeloid leukemia. This malignancy leads frequently to AHSCT. In our sample it was one of the most important risk factors as well as longer hospital stay, the use of haploidentical cells, diuretics and antihypertensive drugs together.

Almost half of the patients urinated on the day of the event from 3.500mL to more than 4.500mL, which resulted in repeated night awakening, most often in conditions of hypotension and fever. Furthermore, noncompliance and incomplete patient self-sufficiency were correlated to these falls.

In AHSCT patients, the conditioning regimen causes myelosuppression through medullary toxicity, as well as nonhematological symptoms such as mucositis, vomiting, diarrhea, pain, fatigue, and mental distress. All these factors may contribute to increase the risk of fall in patients where conditioningrelated thrombocytopenia may lead to severe hemorrhagic complications.^[10]^

Miwa et al^[12]^ described a 30% time of falls in the preengraftment phase. Although no serious injuries were reported, in 66.67% of reported falls, minor head trauma was observed, which has potentially very serious immediate or late consequences. Precisely, possible negative developments of traumas after falls require patient monitoring and care actions aimed at detecting signs and/or early neurological symptoms to implement effective therapeutic interventions.^[23]^ Our results were in line with previous studies^[10,12]^ where no patients reported serious injuries as consequence of fall. This was probably attributable to the timing of the events, which happened on average 24 days after stem cells infusion, when the “engraftment” was already occurred.

Our findings showed longer hospitalizations in patients who fallen. However, considering the mildness of the problems caused by falls to these patients, it is reasonable to assume that other factors concurred to affect their hospital stay. In addition, it is also likely to believe that factors acted prolonging hospital stay have also had any effect on falls.

Another interesting result of our study was that in 80% of patients, fall risk was assessed with the Conley scale - one of the most widely used assessment tools in literature and gradually introduced from 2017 in the context of observed care - in average 6.8 days prior to the event. In our study, we observed a sensitivity of 75% even if not statistically significant, which is related to the small patient cohort. However, our sensitivity is in line (only slightly higher) than in other studies, which reported a sensitivity of 60% to 69% and a specificity of 41% to 61%.^[24-26]^

Although fall risk assessments can have generally beneficial implications involving operators, patients and family/caregivers in the assessment process,^[27]^ Lovallo et al^[28]^ showed that patients not at risk paid less attention during their hospital stay. The fall should be considered as a multifactorial event in which many causes and various aspects inherent in the degree of autonomy of the patient in daily life need to be considered. For this reason, predictive fall risk scales should not be used, which attribute a simple score, particularly in a context of extreme fragility and mutability of clinical conditions such as that of AHSCT. Multidimensional patient assessment tools (neurocognitive area; hemodynamic area; urinary incontinence and use of drug), which integrate with a preventive intervention plan, are preferable.^[29,30]^ Another aspect we consider relevant is that 60% of our falls occurred during night between 10 PM and 7 AM, and on average falls occurred 24 days after stem cells infusion, in the postengraftment phase. This is in line with other literature,^[31,32]^ but not with Ueki et al,^[11]^ who identified for the same stage mainly “daytime” as time of occurrence. However, night-time appears to be particularly risky due to multiple factors: disorientation of the patient, presence of obstacles in an unfamiliar environment, low night brightness of the room, autonomous behavior in an unknown bed, and reduced staffing ratio. In patients at high risk of falling, additional protective measures such as our “Safe Comfort” checklist could reduce this risk, which we implemented at our AHSCT unit. Our study recruited AHSCT patients only due to the lack of information on other hematology settings. However, other patients such as those undergoing autologous HSCT are exposed to many of the fallassociated risk factors descripted above. This could be considered in hematology setting approaching fall prevention.

## 5. Limitation

RCA is a reactive method of analysis, essentially retrospective depending on one or more linear events. Furthermore, this method may result in cognitive biases related to the potential lack of third party involvement. Generally, it is preferable that institutions/hospitals act in a proactive way to detect dangers associated with treatment and to assess risks. Failure mode and effect analysis might be an alternative, which systematically assesses how a process, product or system fails; in particular, it assesses how failure or defect manifests affecting a process.^[33]^ However, we decided for RCA because represents the most effective tool for the analysis of adverse events. Another limitation is the single center experience and small sample size of this study, which does not allow generalization of the results. However, we included all AHSCT falls occurring between December 2017 and November 2019 in our study with the attempt to improve safety further. Furthermore, our analysis is based on a comprehensive and in-depth review of medical records and IRs for each single patient and each expert panel member reviewed these data independently.

## 6. Conclusions

Further studies in the AHSCT setting are necessary to gain more insights on root causes for falls. Haploidentical transplantation, advanced age and acute myeloid leukemia, represent important risk factors related to falls. Moreover, the risk of falling increases the longer a patient is hospitalized. Diuretic drugs inducing hyper-diuresis, partial self-sufficiency, poor patient compliance, orthostatic hypotension, fever, night-time, and obstacles within inpatient units are the most common contributing factors.

We propose to train nurses and to reschedule administration of diuretic and antihypertensive drugs. Despite the prediction tools designed to assess risk in the hospital setting, they have so far proved neither robust nor reliable, it is appropriate use a multidimensional risk assessment scale integrated with a preventive action plan such as the safe comfort checklist to reduce the likelihood of fall recurrences.

## Author contributions

The authors contributed equally.

**Conceptualization:** Marco Cioce.

**Formal analysis:** Marco Cioce, Andrea Bacigalupo.

**Methodology:** Marco Cioce.

**Supervision:** Giuseppe Vetrugno, Paolo Oppedisano, Maurizio Zega, Simona Sica, De Stefano Valerio, Andrea Bacigalupo, Alberto Fiore.

**Validation:** Elena Rostagno, Laura Orlando.

**Writing - original draft:** Marco Cioce.

**Writing - review & editing:** Franziska Michaela Lohmeyer, Stefano Botti, Elena Rostagno, Laura Orlando, Paolo Oppedisano, Alberto Fiore.
